# Urban social determinants of non-communicable diseases risk factors in Argentina

**DOI:** 10.1016/j.healthplace.2021.102611

**Published:** 2022-09

**Authors:** Natalia Tumas, Santiago Rodríguez López, Usama Bilal, Ana F. Ortigoza, Ana V. Diez Roux

**Affiliations:** a Departament de Ciències Polítiques i Socials, Universitat Pompeu Fabra, Barcelona, Spain; b Centro de Investigaciones y Estudios sobre Cultura y Sociedad, Consejo Nacional de Investigaciones Científicas y Técnicas y Universidad Nacional de Córdoba, Argentina; c Facultad de Ciencias Exactas, Físicas y Naturales, Universidad Nacional de Córdoba, Argentina; d Urban Health Collaborative, Dornsife School of Public Health, Drexel University, Philadelphia, PA, USA; e Department of Epidemiology and Biostatistics, Dornsife School of Public Health, Drexel University, Philadelphia, USA

**Keywords:** Social determinants, Non-communicable diseases, Urban, Mixed models, Argentina

## Abstract

We examined associations of individual-, neighborhood- and city-level education -as proxies of SES at different levels-, with diabetes, hypertension, obesity, smoking and binge drinking (non-communicable disease risk factors -NCD/RF) among Argentinian adults. We estimated mixed models based on 21,415 individuals from the 2013 National Survey of Risk Factors, living in 2,698 neighborhoods and 33 cities. Gradients by individual-level education differed by gender and NCD/RF, and some were modified by city education. In addition, we identified contextual effects of neighborhood and city education on some NCD/RF. Urban efforts to tackle NCD/RF in Argentina should be context- and gender-sensitive, and mainly focused on socially disadvantaged groups.

## Highlights

• City-, neighborhood- and individual-level education shape non-communicable diseases risks factors (NCD/RF) in Argentina.
• Most associations across levels vary by gender and NCD/RF.
• Educational gradients are stronger for individual- and neighborhood-level.
• Some of these associations interact with city education.
• Urban public strategies to tackle NCD/RF should be context and gender-sensitive.

## 1 Introduction

More than one-half of the global burden of disease is attributable to non-communicable diseases (NCD) (Benziger et al., 2016). NCD share key modifiable risk factors (RF) like tobacco use, alcohol intake, unhealthy diets, physical inactivity and obesity (WHO, 2017). A number of factors contribute to the NCD/RF burden, including environmental, social, political, and commercial factors (Miranda et al., 2019). Among these, urbanization has emerged as one of the main determinants of the rising burden of NCD/RF (Allender et al., 2008; Flies et al., 2019). The greater access to processed, high calorie, high fat and salty foods as well as the more sedentary lifestyles -due to less physically demanding employment and less active transport that accompanies urbanization-, may contribute to the links between urban living and NCD/RF (Allender et al., 2008; Ruel et al., 2017).

Among urban populations socioeconomic status (SES) is known to be strongly associated with NCD/RF in high-income countries (Diez Roux et al., 2000; Marmot and Bell, 2019). Although some evidence from low- and middle-income countries (LMICs) also shows similar gradients (Williams et al., 2018; Allen et al., 2017; Hosseinpoor et al., 2012), empirical studies are more limited and also suggest that important heterogeneities in the social patterning of NCD/RF may be present (Fleischer et al., 2011a; Sommer et al., 2015; Jiwani et al., 2019). Over and above the role of individual SES, area-level or neighborhood SES is also associated with NCD/RF (Pi et al., 2018; Bilal et al., 2018a, 2019; Corriere et al., 2014). Indeed, evidence from experimental studies suggests that moving from a neighborhood with higher SES to one with lower SES leads to reductions in extreme obesity and diabetes (Ludwig et al., 2011). Area or neighborhood-level factors may affect health through several mechanisms including effects on health behaviors and stress-related processes, independently of individual-level SES (Diez Roux, 2018; Christine et al., 2017; Pardo-Crespo et al., 2013; Diez Roux and Mair, 2010).

The social features of cities themselves may also affect NCD/RF risk directly or may buffer or accentuate social patterning by individual- or neighborhood-level SES (Goryakin et al., 2017; Diez Roux, 2015; Wang et al., 2018). For instance, in the context of higher city SES, differences in NCD/RF by individual education may be smaller because of favorable effects of higher city SES especially among individuals with lower SES, who may be more dependent on city services and more affected by city environments. Similarly, in cities with higher SES neighborhood SES differences may be smaller because neighborhoods of lower SES may benefit from the resources available in higher SES cities such as better access to healthy foods or health care or improved transport infrastructure which may facilitate access to resources in other parts of the city. Conversely, lower city SES may result in larger differences by education at the individual or neighborhood-level because of more limited access to city resources and weaker social protections for the poor (Stafford and Marmot, 2003; Luo et al., 2019), and also because social interactions and opportunities might be more restricted to the neighborhood of residence (Wang et al., 2018).

Given that urbanization can exacerbate poverty and social inequalities (Liddle, 2017), it is especially important to examine social differences in NCD/RF within urban settings. Understanding how city-, neighborhood- and individual-level factors jointly shape the social patterning of NCD/RF is critical to urban and health policies to reduce inequities. A major evidence gap is present in middle-income highly urbanized countries, such as Argentina, that are increasingly experiencing the adverse health impacts of NCD/RF. We used city-, neighborhood- and individual-level data compiled by the SALURBAL study (Diez Roux et al., 2018; Quistberg et al., 2019) for Argentina to examine how individual-, neighborhood-, and city-level SES are associated with NCD/RF among urban adults. We hypothesized that lower individual-, neighborhood-, and city-level SES would be independently associated with higher prevalence of NCD/RF. We also hypothesized that differences by individual- and neighborhood-level SES on NCD/RF would be greater in cities with lower SES than in those with higher SES.

## 2 Material and methods

### 2.1 Data

Data was compiled as part of the SALURBAL project, which identified cities with 100,000 inhabitants or more in 2010 (Diez Roux et al., 2018; Quistberg et al., 2019). Cities were defined as clusters of administrative units that encompassed the visually apparent urban agglomerations as identified using satellite images. In Argentina, neighborhoods within cities were proxied by ‘Radios Censales’, spatially delimited units used for census data collection. These small census tracts have a population median (5th-95th percentile) of 883 (250–1692), and a median of approximately 300 households (for details see Quistberg et al., 2019). Individual-level data came from the 2013 National Survey of Risk Factors of Argentina (‘Encuesta Nacional de Factores de Riesgo’, ENFR), which has a cross-sectional probabilistic multistage sampling design, with provincial and nationally representative sample (INDEC, 2015). City- and neighborhood-level data were obtained from the 2010 National Census of Argentina (INDEC, 2012).

The final sample comprised 21,451 individuals over 18 years old (56.2% women) living in 2,698 neighborhoods and 33 cities (each neighborhood is nested in a city, for more details please see Quistberg et al., 2019). There was a median of 9 survey respondents per neighborhood (interquartile range (IQR): 7–11), 83 neighborhoods per city (IQR: 61–132), and 693 survey respondents per city (IQR: 499-1,115). A map with the cities included in the study is provided in the supplementary material (Fig. S1).

### 2.2 Individual- and area-level exposures

We included three exposure variables as proxies of SES at different levels: i) individual-level education: individual education (less than primary, primary, secondary, and university); ii) neighborhood-level education: the proportion of the population aged 25 or older who completed secondary education or above; and iii) city-level education: the proportion of the population aged 25 or older who completed secondary education or above. Both neighborhood and city education were standardized in regression analysis.

### 2.3 Outcomes

We evaluated five dichotomous outcomes at the individual level: a. Diabetes, defined as the presence of diabetes diagnosed by a healthcare provider among all adults (excluding diagnosis during pregnancy); b. Hypertension, defined as the presence of hypertension diagnosed by a healthcare provider among all adults (excluding diagnosis during pregnancy); c. Obesity, defined as body mass index was 30 kg/m^2^ or higher based on self-reported height and weight; d. Smoking, defined as current cigarette smokers; e. Binge drinking, defined as 3 or more alcoholic drinks for women and 4 or more alcoholic drinks for men on at least one occasion in the past 30 days. All outcomes (including diagnoses by a healthcare provider) were self-reported.

### 2.4 Other covariates

Analyses were adjusted by survey respondent's age, in order to account for different age structures. We included cities' population size as a potential confounder, since previous studies have reported that living in larger cities was associated with lower probability of NCD/RF (Rocha et al., 2015) and with greater poverty (Liddle, 2017).

### 2.5 Statistical analysis

We first described the distribution of individual and contextual characteristics by presence or absence of NCD/RF. We also estimated age adjusted prevalence of NCD/RF by levels of individual, neighborhood, and city education. We used gender-stratified three-level logistic mixed models, with individuals nested in neighborhoods nested in cities, to estimate odds ratios of NCD/RF associated with predictors of interest. For each dichotomous outcome, we first fitted a model with no explanatory variables and a random effect at the neighborhood- and city-level (empty model). The adjusted model added individual, neighborhood and city education, age and city population size. Finally, interaction models a and b included interaction terms to examine whether city-level education modifies the associations between individual-level education and NCD/RF (interaction model a) or between neighborhood-level education and NCD/RF (interaction model b). We standardized continuous variables in all models (centered by their mean and scaled by their standard deviation).

All models were fitted using the following general equation (corresponding to the adjusted model):

$$logit\;of\;P_{ijk}=\gamma_{000}+\gamma_{100}Ind.Edu_{ijk}+\;\gamma_{200}Age_{ijk\;}+\;\gamma_{010}Neigh.Edu_{jk}\;+\gamma_{001}City.Edu_{k}+\gamma_{002}City.Pop_{k}+\;\vartheta_{jk}+\vartheta_{k}$$

where $P_{ijk}$ is the probability for each NCD/RF in the *ith* individual, living in the *jth* neighborhood and the *kth* city; $\gamma_{100}$ is the estimate coefficient of individual education; $\gamma_{200}$ is the estimate coefficient of age; $\gamma_{010}$ is the estimate coefficient of the neighborhood education; $\gamma_{001}$ is the estimate coefficient of city education; $\gamma_{002}$ is the estimate coefficient of the total population at the city level; $\vartheta_{jk}$ is the random intercept for neighborhoods and $\vartheta_{k}$ is the random intercept for city, both normally distributed with variances $\tau_{00}^{2}$ and $\tau_{0}^{2}$.

Statistical analyses (under a frequentist framework) were performed using Stata 14 and the *melogit* command. Global p-values for interaction were obtained using the *testparm* command. Global p-values reflect improvement in model fit using likelihood ratio tests when all interaction terms (reflecting interactions between categories) are added together. Given that our intent was to estimate associations between SES variables and NCD/RF, rather than estimate population's prevalence of NCD/RF, no weights were included in analyses (Diez Roux et al., 2000). However, variables relevant to weights such as age and gender were included for adjustment.

## 3 Results

Table 1 shows individual-, neighborhood- and city-level characteristics by NCD/RF. Respondents who reported having hypertension were more likely to be women than those who did not. In contrast, respondents who reported smoking and binge drinking were more likely to be male than those who did not. Individuals with diabetes, hypertension and obesity were older than those who did not report these conditions, while those who reported smoking or binge drinking habits were younger than those who did not. Overall, lower educational levels were associated with higher likelihood of reporting diabetes, hypertension and obesity. Educational differences were smaller and less consistent for binge drinking and smoking although persons who reported these habits were more likely to have primary education only compared to those who did not. Additionally, respondents with hypertension, obesity or smoking habits were more likely to live in neighborhoods with lower education than those who did not report these conditions. Overall, and despite some statistically significant differences, there seemed to be no meaningful differences in city education when comparing individuals with and without NCD/RFs (Table 1).

**Table 1:** Individual-, neighborhood- and city-level characteristics by non-communicable disease risk factors. Argentina, National Survey of Risk Factors, 2013 (n = 21,451).

	Diabetes		Hypertension		Obesity		Smoking		Binge drinking	
	Yes (n = 1,852)	No (n = 19,485)	Yes (n = 6,956)	No (n = 14,330)	Yes (n = 5,516)	No (n = 15,935)	Yes (n = 5,755)	No (n = 15,696)	Yes (n = 4,280)	No (n = 16,984)
Women, %	54.37	56.37	61.17	53.98	55.13	56.55	48.27	59.09	37.83	60.61
*p value* (χ^2^)	*0.098*		*< 0.001*		*0.066*		*< 0.001*		*< 0.001*	
Age, mean (SD)	56.95 (16.22)	43.57 (17.70)	54.33 (17.70)	40.16 (16.19)	48.27 (17.18)	43.52 (18.08)	40.38 (14.77)	46.34 (18.77)	36.26 (14.19)	46.92 (18.20)
*p value* (*t-test*)	*< 0.001*		*< 0.001*		*< 0.001*		*< 0.001*		*< 0.001*	
Individual education, %										
Less than primary	16.95	8.57	14.32	6.88	14.41	7.56	7.91	9.84	6.31	10.11
Primary	41.52	36.43	42.88	33.90	43.49	34.64	42.42	34.90	39.46	36.33
Secondary	28.19	37.81	28.98	40.82	30.84	39.03	36.92	36.93	40.49	35.92
University	13.34	17.19	13.82	18.40	11.26	18.77	12.75	18.34	13.74	17.64
*p value* (χ^2^)	*< 0.001*		*< 0.001*		*< 0.001*		*< 0.001*		*< 0.001*	
Neighborhood education, mean (SD)	46.81 (19.58)	47.71 (20.58)	46.87 (19.88)	48.05 (20.79)	43.73 (19.21)	48.98 (20.76)	45.84 (20.19)	48.29 (20.58)	47.49 (21.3)	47.67 (20.31)
*p value* (*t-test*)	*0.071*		*< 0.001*		*< 0.001*		*< 0.001*		*0.611*	
City education, mean (SD)	38.14 (3.39)	38.00 (3.46)	37.98 (3.56)	38.03 (3.40)	38.00 (3.44)	38.02 (3.46)	38.14 (3.36)	37.96 (3.49)	38.24 (3.34)	37.95 (3.48)
*p value* (*t-test*)	*0.109*		*0.334*		*0.725*		*< 0.001*		*< 0.001*	

p-values test the null hypothesis that there is no difference in the proportion (χ^2^) or mean (*t*-test) of the characteristic between people with and without the NCD/RF.

**Fig. 1:**
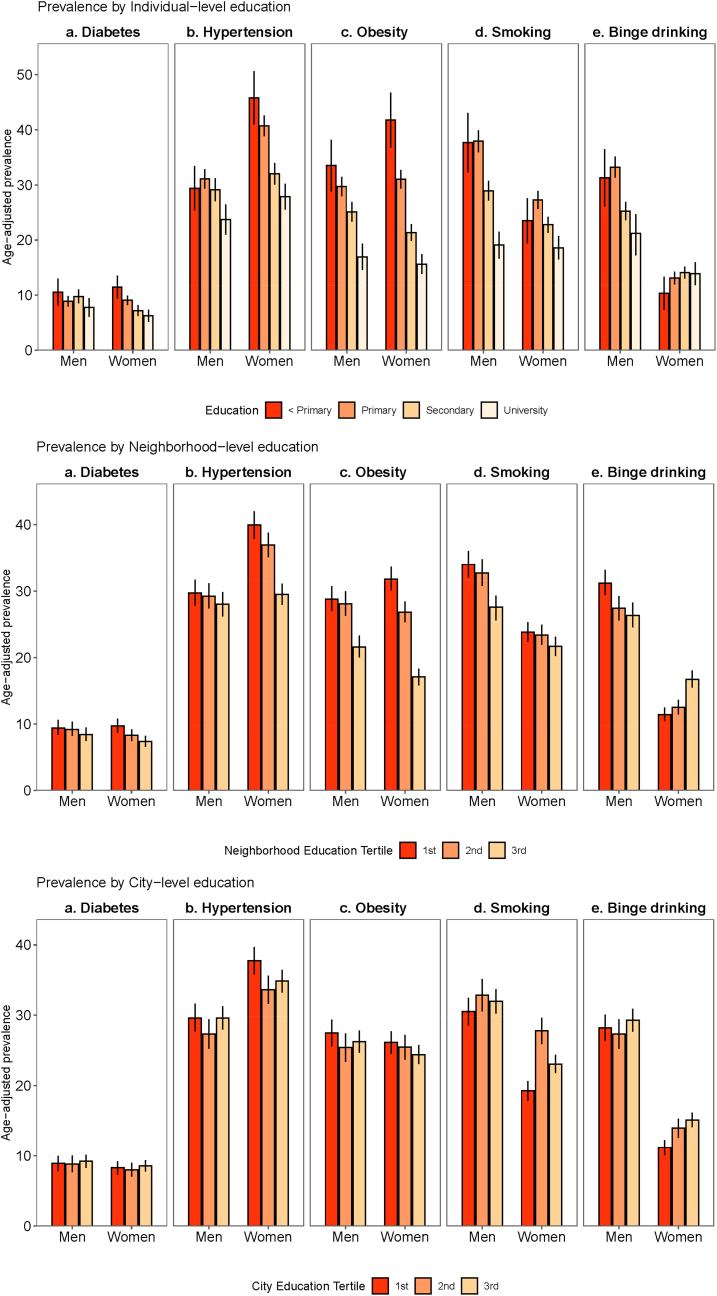
Age-adjusted prevalence (95% confidence intervals) of non-communicable disease risk factors by a) individual-, b) neighborhood- and c) city-level education. Argentina, National Survey of Risk Factors, 2013. Age-adjusted using the age distribution of the sample. 95% confidence intervals are shown in vertical lines.

Fig. 1 shows the age-adjusted prevalence of NCD/RF by individual-, neighborhood-, and city-level education. The prevalence of obesity, hypertension and diabetes by individual education exhibited a social gradient, with higher prevalence in people with lower levels of education, especially among women. While binge drinking and smoking prevalence were more frequent among men with lower education than in those with higher education, in women, binge drinking was more common among those with higher education and smoking exhibited a slight downward trend as education increased from primary to upper levels (Fig. 1a). We also observed a social gradient in the prevalence of NCD/RF by neighborhood education (Fig. 1b). Differences by neighborhood-education were small for diabetes in both genders. Women living in neighborhoods with higher education had lower prevalence of hypertension and obesity, and higher prevalence of binge drinking. Women's smoking prevalence was similar across all neighborhood education categories. Among men, obesity, binge drinking, and smoking were higher in neighborhoods with lower education, while the social gradient in hypertension was weaker. Overall, the social patterning of NCD/RF at the city level was less evident (Fig. 1c). No differences by city education were observed for diabetes or obesity in both genders. Women living in cities with lower education had higher prevalence of hypertension and lower prevalence of binge drinking and smoking than women living in cities with higher education, but we found no differences among men (Fig. 1).

Table 2 shows associations of NCD/RF with individual, neighborhood and city education. In general, there was more variability in outcomes across neighborhoods within cities than between cities (see variance components in Table 2). In men, there were clear gradients in obesity and smoking (and weaker and less consistent gradients for hypertension and binge drinking) associated with individual education, with higher odds of NCD/RF in those of lower educational level. Diabetes was not associated with education. We also found that men living in neighborhoods with higher education had lower odds of obesity (OR per SD higher neighborhood education 0.87, 95% CI 0.82 to 0.92). There were no significant associations between neighborhood education and hypertension, diabetes, smoking, or binge drinking. City education was not associated with NCD/RF (Table 2).

**Table 2:** Odds ratios of non-communicable disease risk factors associated with individual, neighborhood, and city education. Argentina, National Survey of Risk Factors 2013 (n = 21,451).

		Diabetes	Hypertension	Obesity	Smoking	Binge drinking
		OR (95% CI)				
Men		(n = 9,346)	(n = 9,295)	(n = 9,398)	(n = 9,398)	(n = 9,351)
	Individual education					
	University	1.00	1.00	1.00	1.00	1.00
	Secondary complete	1.19 (0.93, 1.54)	1.26 (1.07, 1.49)	1.35 (1.14, 1.60)	1.34 (1.14, 1.57)	1.12 (0.95, 1.33)
	Primary complete	1.06 (0.82, 1.38)	1.42 (1.20, 1.69)	1.55 (1.30, 1.84)	1.99 (1.69, 2.35)	1.73 (1.45, 2.06)
	Less than primary	1.07 (0.77, 1.49)	1.18 (0.94, 1.48)	1.62 (1.29, 2.02)	1.88 (1.51, 2.33)	1.59 (1.24, 2.02)
	Neighborhood education, z-score	0.93 (0.85, 1.02)	0.99 (0.93, 1.05)	0.87 (0.82, 0.92)	0.97 (0.92, 1.02)	0.98 (0.92, 1.04)
	City education, z-score	1.06 (0.94, 1.20)	1.03 (0.95, 1.11)	1.01 (0.91, 1.10)	1.05 (1.00, 1.10)	1.09 (0.78, 1.01)

	City variance (Std. error)	0.065 (0.034)	0.021 (0.013)	0.029 (0.014)	0.004 (0.007)	0.130 (0.041)
	Neighborhood variance (Std. error)	0.297 (0.110)	0.159 (0.053)	0.160 (0.047)	0.059 (0.039)	0.313 (0.058)

Women		(n = 11,991)	(n = 11,991)	(n = 12,053)	(n = 12,053)	(n = 11,913)
	Individual education					
	University	1.00	1.00	1.00	1.00	1.00
	Secondary	1.03 (0.83, 1.29)	1.15 (1.01, 1.30)	1.18 (1.02, 1.36)	1.16 (1.02, 1.32)	1.11 (0.95, 1.30)
	Primary	1.27 (1.02, 1.58)	1.62 (1.42, 1.84)	1.70 (1.47, 1.96)	1.50 (1.30, 1.72)	1.20 (1.01, 1.44)
	Less than primary	1.54 (1.18, 2.02)	1.81 (1.52, 2.16)	2.46 (2.04, 2.96)	1.11 (0.91, 1.37)	0.95 (0.70, 1.28)
	Neighborhood education, z-score	0.88 (0.81, 0.95)	0.86 (0.81, 0.90)	0.73 (0.69, 0.77)	0.99 (0.94, 1.04)	1.27 (1.19, 1.35)
	City education, z-score	1.12 (1.02, 1.23)	0.97 (0.90, 1.06)	1.10 (0.99, 1.22)	1.08 (0.98, 1.19)	1.09 (0.96, 1.23)

	City variance (Std. error)	0.022 (0.018)	0.039 (0.015)	0.074 (0.025)	0.065 (0.022)	0.093 (0.033)
	Neighborhood variance (Std. error)	0.153 (0.088)	0.111 (0.036)	0.145 (0.039)	0.037 (0.037)	0.228 (0.065)

Multilevel structure: individuals nested within neighborhoods, nested within cities. Analyses adjusted by age and total city population. Neighborhood education: proportion of the population aged 25 or older who completed secondary education or above. City education: proportion of the population aged 25 or older who completed secondary education or above. The estimates correspond to the adjusted model. Std. error corresponds to the standard error of the random component of cities and neighborhoods.

In women, lower individual-level education was associated with higher odds of diabetes, obesity and hypertension. The social patterning of smoking and binge drinking was less consistent: women with primary and secondary education had higher odds of smoking, and those with primary education had higher odds of binge drinking. These associations were much weaker than those evidenced in men, and we observed no significant association of smoking or binge drinking with less than primary education. In addition, higher neighborhood education was associated with lower odds of diabetes (OR 0.88, 95% CI 0.81 to 0.95), hypertension (OR 0.86, 95% CI 0.81 to 0.90) and obesity (OR 0.73, 95% CI 0.69 to 0.77), and with higher odds of binge drinking (OR 1.27, 95% CI 1.19 to 1.35). Higher city education was only associated with higher odds of diabetes (OR 1.12, 95% CI 1.02 to 1.23) (Table 2).

**Fig. 2:**
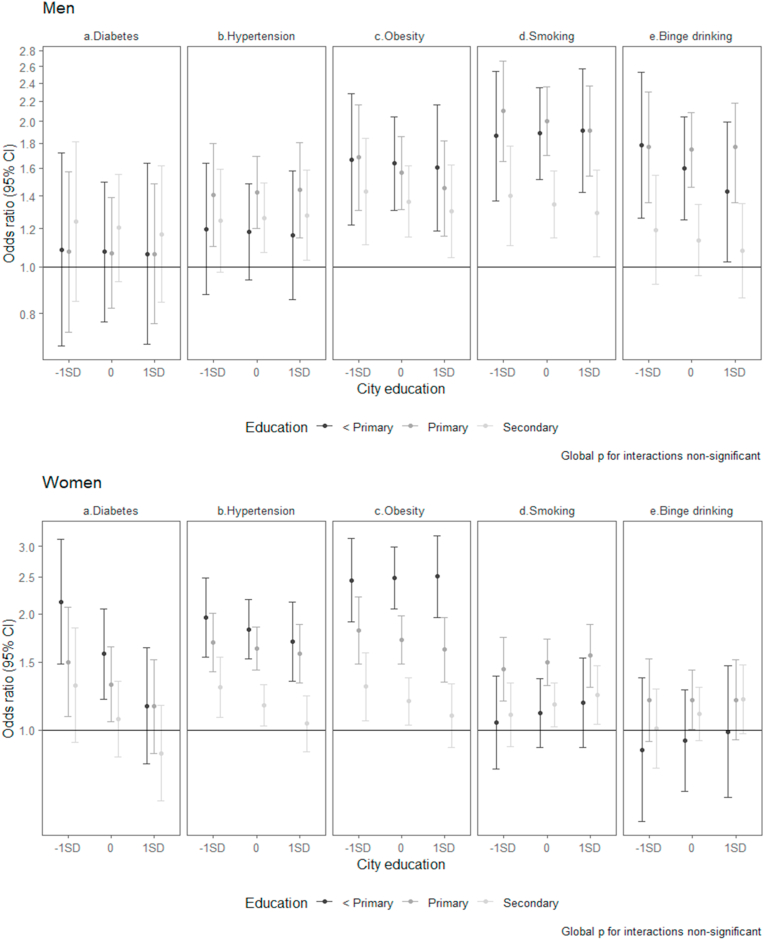
Adjusted odds ratios of non-communicable disease risk factors associated with individual-level education stratified by city education as estimated from multilevel models.

Fig. 2 shows adjusted odds ratios for individual education by levels of city education. Interactions between individual and city education were not statistically significant for either gender. However, point estimates showed that in women the odds ratios of diabetes and hypertension associated with lower education were of smaller magnitude in higher education cities than in lower education cities. For example, the higher odds of diabetes in women with less than primary education, as compared to women with university education, were reduced by 27% for each one-SD increase in city education. In contrast, in women the odds ratios of smoking associated with lower education were slightly higher in higher education cities compared to lower education cities. In men, the increased odds of binge drinking in men with less than primary education, as compared to men with university education, decreased by 11% in cities with higher education (Fig. 2).

**Fig. 3:**
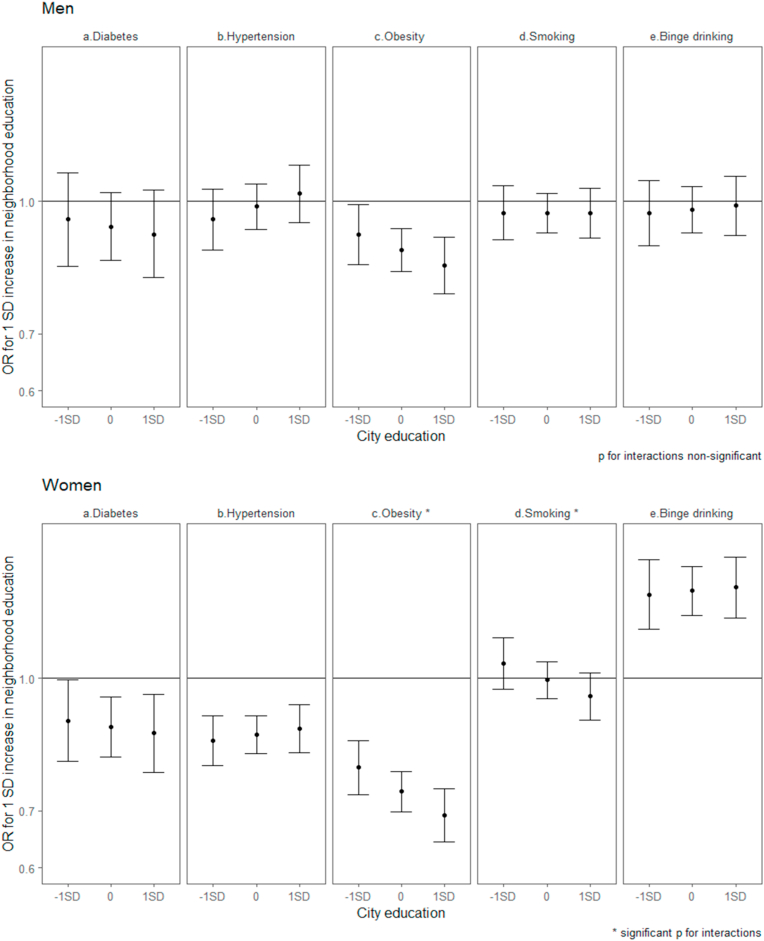
Adjusted odds ratios of non-communicable disease risk factors associated with neighborhood-level education stratified by city education as estimated from multilevel models.

Fig. 3 shows adjusted odds ratios for neighborhood education by levels of city education. We found no statistically significant interaction in men, whereas in women there were significant interactions for obesity and smoking. The association of higher neighborhood education with lower odds of obesity were stronger in higher SES cities than in lower SES cities (Fig. 3). A similar albeit weaker pattern was observed in men. In women, higher neighborhood SES was associated with higher odds of smoking in lower SES cities but with lower odds of smoking in higher SES cities. Estimates from models with interactions are shown in supplementary tables (supplementary material, Tables S1 and S2).

## 4 Discussion

We investigated associations between proxies of SES at different levels and several NCD/RF in Argentinean cities. Patterns of social gradients across levels of analysis differed by gender and NCD/RF. We identified an inverse association between individual education and all NCD/RF except for diabetes in men. However, the gradients were less clear for binge drinking and smoking in women. We also found that after accounting for individual education, higher neighborhood education was associated with lower odds of obesity in both genders, and with lower odds of diabetes and hypertension in women. In contrast, in women higher neighborhood education was associated with higher odds of binge drinking. Higher city education was associated with higher odds of diabetes among women but no other associations with city education were observed.

Results also suggested that city education may modify associations of individual and neighborhood education with NCD/RF. In women the inverse association of individual-level education with diabetes and hypertension were of smaller magnitude in higher education cities than in lower education cities, but the opposite pattern was observed for smoking. In men, the inverse associations of individual education with binge drinking in men were also of smaller magnitude in higher SES cities. City SES also appeared to modify neighborhood differences: the inverse association of neighborhood education with obesity was stronger in higher SES cities than in lower SES. In women, higher neighborhood SES was associated with higher odds of smoking in lower SES cities but with lower odds of smoking in higher SES cities.

We found that lower individual-level education was associated with higher odds of hypertension and obesity in both genders, and with higher odds of diabetes only in women. Compelling evidence supports that lower SES is linked to a higher risk of NCD (Niessen et al., 2018; McNamara et al., 2017; Di Cesare et al., 2013; Lago-Peñas et al., 2020). However, evidence on the association between individual SES and obesity and diabetes is mixed, and patterns may differ (Williams et al., 2018; Lago-Peñas et al., 2020; Jiwani et al., 2019): as countries develop and the nutrition transition progresses, the burden shifts from higher to lower SES groups (Jones-Smith et al., 2012; Monteiro et al., 2004a, 2004b; Popkin, 2015). Research from some LMICs suggested that higher individual SES was related to higher diabetes risk (Williams et al., 2018). However, most studies from upper middle- and high-income countries reported increased risk of diabetes in lower SES groups (Lago-Peñas et al., 2020). In addition, previous research showed that hypertension and body mass index were inversely associated with education in middle- and high-income countries (Di Cesare et al., 2013). The social patterning of obesity and diabetes was previously reported in Argentina (Linetzky et al., 2013; Ferrante et al., 2016; Fleischer et al., 2011a). Aligned with our results, Argentina fits a pattern in which obesity burden is concentrated in lower SES groups (Jiwani et al., 2019), which is also consistent with the stage 3 of the obesity transition, characterized by a higher prevalence in lower SES groups (Jaacks et al., 2019). Overall, our findings on individual education and hypertension, obesity and diabetes (only in women) are similar to those reported from upper-middle and high-income countries.

Research shows that individuals with lower education are more likely to adopt behaviors that increase risk of NCD, such as tobacco use and binge drinking (Borges et al., 2016; Lim et al., 2012; Lago-Peñas et al., 2020; Hiscock et al., 2012). Prior work reported higher prevalence of harmful drinking and tobacco use in lower SES groups (Allen et al., 2017; Bilal et al., 2016; Faggiano et al., 2001; Hiscock et al., 2012). Educational gradients in smoking were also observed in Argentina (Ferrante et al., 2016; Fleischer et al., 2011b; Santero et al., 2019). We also found that lower education was linked to higher probability of smoking and hazardous drinking, with stronger associations among men. However, there is also evidence showing that higher education was associated with more smoking in women from southern Europe (Huisman et al., 2005; Santos and Barros, 2004). This was also described for older women in Argentina (Fleischer et al., 2011b). Among the mechanisms behind the links between SES and health outcomes, it was proposed that opportunities to adopt healthy behaviors are less available among individuals living in poverty and this result in a higher probability of NCD/RF (Wagstaff, 2002). In addition, the educational background influences the individuals' receptivity to health education messages (Batis et al., 2019). A reverse pathway may also exist, since NCD/RF leads to higher expenditures and reduced income status (Jan et al., 2018; Niessen et al., 2018). Moreover, evidence suggests that excessive alcohol consumption lowers cognitive bandwidth which results in worse economic consequences (Schilbach et al., 2016).

We also found gender differences in educational gradients of NCD/RF: while for diabetes, hypertension and obesity the educational patterning was more clearly defined in women, for smoking and binge drinking it was clearer among men. Previous work also documents different associations by gender. Jiwani et al. (2019) highlighted that obesity was more prevalent among women with lower SES in several Latin American countries, including Argentina. Other work reported that Argentinian women from lower SES (lowest wealth tertile) had a higher prevalence of obesity, compared with their counterparts from higher SES (Batis et al., 2019). Moreover, a recent study in Latin America showed higher obesity risks for lower educated women regardless of city SES, while among men education attainment was directly associated with obesity in lower city SES but inversely associated in wealthier cities (Mazariegos et al., *in press*). Explanations for the gender disparities in the educational gradients in diabetes, hypertension and obesity found in our study might include the more physically demanding jobs for men with lower education, and the gendered social norms about physical features that are considered desirable for men and women (McLaren et al., 2009). Further, in a patriarchal system women's appearance is highly emphasized, with heteronormative gender norms often shaping more educated women's behaviors (e.g. they are more likely to face pressure to change their bodies to align social expectations) (Heise et al., 2019).

We did not find a consistent social patterning for health behaviors like smoking and binge drinking among women. Studies in high income countries have showed that the social patterning of smoking was more consistent among men (Huisman et al., 2005). In Spain, as smoking prevalences in women and men have converged, there has been a trend towards higher prevalence of smoking in lower SES women (Bilal et al., 2016). Previous work in Argentina in 2005 showed a linkage between higher SES and lower smoking in men and in younger women, but to higher smoking in older women (Fleischer et al., 2011b). Recently, another study in Argentina described higher smoking prevalence for lower educational levels in men, while this association was less evident among women (Santero et al., 2019). Possible reasons to explain the gender-specific associations in the behavioral risk factors include that men are more likely to respond to stress by externalizing behaviors through binge drinking (Matheson et al., 2012), as well as the different social norms in women and men (for example, smoking may be perceived as a symbol of status or independence in higher SES women). The absence of a consistent social patterning for unhealthy behaviors may suggest that Argentina might be experiencing a change in the educational patterning of these behaviors, especially among women.

Our study also showed interesting neighborhood and city contextual effects. After accounting for individual education, we found that among women, higher neighborhood education was linked to lower odds of diabetes, hypertension and obesity, but to higher odds of binge drinking. Among men, higher neighborhood education was associated with lower odds of obesity. Aligned with our results for women, earlier research reported that individuals living in higher SES neighborhoods had lower mean blood pressure (Kivimäki et al., 2018), which was particularly related to the neighborhood food environment (Kaiser et al., 2016). Also similar to our findings, other studies reported lower body mass index in neighborhoods with higher education (Mujahid et al., 2005; Ross et al., 2007), independent of individual education. Moreover, lower prevalence of diabetes was reported in neighborhoods with higher SES (Bilal et al., 2018b), with this association being stronger among women. Higher prevalence of diabetes was also reported among African American women living in neighborhoods with lower SES (Krishnan et al., 2010). Results from prospective studies (Kivimäki et al., 2018; Krishnan et al., 2010) also support similar associations. The underlying mechanisms behind this association could be related with lower availability of healthy foods or physical activity promoting resources in neighborhoods (Auchincloss et al., 2009). Women living in neighborhoods with higher education had greater probability for binge drinking. Contrary to our results, Fone et al. (2013) found that binge drinking was more common in deprived areas. Higher city SES was associated with more diabetes after adjusting for neighborhood and individual-level SES. Additional research is needed to better understand the factors diving in these contextual associations.

We also documented some differences by city education in the associations between NCD/RF and individual or neighborhood education. Overall, higher city education appeared to mitigate the inverse associations of individual level education with diabetes and hypertension in women and with binge drinking in men, and further exacerbate the negative associations between neighborhood education and obesity. A recent study in Latin American cities documented that city SES modified the association between individual education and obesity in men, changing from a direct association in lower city SES to an inverse association in higher city SES (Mazariegos et al., *in press*). In Brazil, an inverse educational gradient for obesity was found only in areas with higher SES for men, while among women the inverse gradient was consistently observed across all SES areas (Monteiro et al., 2001). Some theoretical models were proposed to explain how area SES interacts with individual SES in regards to health outcomes. The ‘double jeopardy’ hypothesis, the collective resources model and the fundamental cause theory overall posit that living in areas with lower SES might be detrimental for the health of individuals with lower SES, since it is hypothesized that they are more dependent on collective resources (Stafford and Marmot, 2003; Luo et al., 2019; Robert, 1999).

Some of these mechanisms might explain the interaction between city education and individual educational differences in NCD/RF found in our study. For example, in cities with better social environments the smaller differences by education might be related to stronger social protection available for more disadvantaged individuals, and the benefits of living in cities with higher SES may be greater for those less educated. In addition, cities with higher SES may have better infrastructure and conditions to connect neighborhoods, and neighbors from more disadvantaged areas could have more opportunities to interact outside their neighborhood (Wang et al., 2018). In contrast to results observed for hypertension and diabetes, in women we also found that the negative association between individual lower education and smoking in women became stronger as city education increased. It is possible that the tobacco advertising images linking smoking with freedom and empowerment (Amos and Haglund, 2000) might be more effective in targeting women living in higher city SES that usually experienced faster gender roles change. However, contrary to our hypotheses, we found that the inverse association of neighborhood education with obesity was stronger in higher SES cities than in lower SES cities, and in women higher neighborhood SES was associated with higher odds of smoking in lower SES cities but with lower odds of smoking in higher SES cities.

A limitation of this study is its cross-sectional design and the possibility of residual confounding. Another limitation is the reliance on self-reported risk factors, which likely introduced measurement error and could be affected by access to care which may differ by SES. Survey response rates could be related to social differences (with higher non-response rates among lower area- and individual-SES), which might lead to an underrepresentation of those groups, as well as to an underestimation of SES differences if response is also linked to the outcomes. We recognize the importance of using different SES variables or composite indicators as proxies of SES, however we selected education because based on past work (Williams et al., 2018; Diez Roux et al., 2007) it seemed to be a reasonable proxy for individual- and area-level SES and could be calculated at all three levels allowing for estimation of contextual effects. In addition, we are also aware that several other factors related to NCD/RF such as physical activity and diet were not included. Nevertheless, most of them are likely to be mediators of the associations of individual and contextual education with the outcomes, and the purpose of our study was not to analyze mediators. Major strengths of our study include the unique dataset and the linkage to harmonized neighborhood and city education indicators. No prior study in Argentina has acknowledged the importance of neighborhood education at a large urban scale, nor analyzed the interplay between proxies of SES at different levels and the NCD/RF profile.

Overall, our findings highlight the need for public strategies aimed to assess social and gender disparities on NCD/RF in Argentina. Some initiatives may include addressing the social determinants themselves, such as tackling poverty or improving access to education among lower SES groups. More targeted interventions to strengthen more disadvantaged groups, such as health education, conditional cash transfers, neighborhood or city-based healthy lifestyles programs. Other interventions targeted at creating healthy neighborhood or city level environments such as improving facilities to access healthy food and regular physical activity, walkability, green spaces, regulations and fiscal measures (e.g. taxes on high fat and sugar foods, alcohol and tobacco) may also contribute. Interventions should take into account the gender norms that underlie NCDs and related risk behaviors.

In summary, our study identified multilevel educational characteristics associated with several NCD/RF, and provided evidence on how these associations varied by gender in urban settings of Argentina. Moreover, our study showed how proxies of SES at different levels interact to shape the social pattern of NDC/RF. Considering the ongoing urbanization process of Argentina, urban efforts to tackle NCD/RF should be context and gender-sensitive, and focused on most disadvantaged social groups.

## Funding

This work was partially supported by the SALURBAL (‘Salud Urbana en América Latina’) project, funded by the 10.13039/100010269Wellcome Trust [205177/Z/16/Z]. This work has received funding from the 10.13039/100010661European Union's Horizon 2020 research and innovation programme under the Marie Sklodowska-Curie grant agreement N° 89102 (Dr. Tumas was supported by this grant). Dr. Bilal was supported by the Office of the Director of the 10.13039/100000002National Institutes of Health under award number DP5OD26429. Dr. Rodríguez López was supported by the 10.13039/501100003074‘Agencia Nacional de Promoción Científica y Tecnológica’ (ANPCyT) of Argentina, Project ‘PICT CONICET 2018-01395’. The funders had no role in the design, analysis or writing of this article.

## Ethics approval

The study protocol has been approved by the Drexel University Institutional Review Board with ID #1612005035.

## Authorship contributions

NT helped conceived and designed the study, conducted the statistical analysis and drafted the manuscript; SRL helped conceived and designed the study, assisted with the statistical analysis and contributed to draft the manuscript; UB assisted with the statistical analysis, and revised critically the manuscript; AO revised critically the manuscript; ADR conceived and designed the study, contributed to the statistical analysis and revised critically the manuscript.

## Declaration of competing interest

None.
