# Supplementary material for: Urban social determinants of non-communicable diseases risk factors in Argentina
Source: Health Place. Author manuscript; Available in PMC 2022 Sep 22. (PMC8714870; doi:10.1016/j.healthplace.2021.102611)
Supplement: Supplementary file 1 [file EMS131095-supplement-Multimedia_component_1.docx]

**Supplementary material**

Figure S1. Argentinian cities included in the study by population size. 

Table S1. Odds ratios of non-communicable disease risk factors by individual and neighborhood education and city education. Argentina, National Survey of Risk Factors 2013 (n= 21,451); interaction model a. 

|  |  | **Diabetes** | **Hypertension** | **Obesity** | **Smoking** | **Binge drinking** |
| --- | --- | --- | --- | --- | --- | --- |
|  |  | **OR (95% CI)** |  |  |  |  |
| Men |  | *(n = 9,346)* | *(n = 9,295)* | *(n = 9,398)* | *(n = 9,398)* | *(n = 9,351)* |
|  | Individual education |  |  |  |  |  |
|  | University | 1.00 | 1.00 | 1.00 | 1.00 | 1.00 |
|  | Secondary complete | 1.20 (0.93, 1.55) | 1.26 (1.07, 1.49) | 1.36 (1.15, 1.61) | 1.34 (1.14, 1.57) | 1.13 (0.95, 1.34) |
|  | Primary complete | 1.06 (0.82, 1.39) | 1.42 (1.20, 1.69) | 1.56 (1.31, 1.86) | 2.00 (1.69, 2.35) | 1.74 (1.45, 2.08) |
|  | Less than primary | 1.07 (0.77, 1.49) | 1.17 (0.93, 1.48) | 1.63 (1.30, 2.04) | 1.88 (1.51, 2.35) | 1.25 (1.25, 2.04) |
|  | Neighborhood education, z-score | 0.93 (0.85, 1.02) | 0.99 (0.93, 1.05) | 0.87 (0.82, 0.92) | 0.99 (0.94, 1.04) | 0.98 (0.92, 1.04) |
|  | City education, z-score | 1.08 (0.85, 1.35) | 1.02 (0.88, 1.19) | 1.07 (0.91, 1.25) | 0.96 (0.91, 1.02) | 1.12 (0.92, 1.37) |
|  | City education*Individual education |  |  |  |  |  |
|  | City education*University | 1.00 | 1.00 | 1.00 | 1.00 | 1.00 |
|  | City education*Secondary complete | 0.97 (0.76, 1.23) | 1.01 (0.86, 1.08) | 0.95 (0.80, 1.12) | 0.95 (0.81, 1.12) | 0.95 (0.80, 1.13) |
|  | City education*Primary complete | 0.99 (0.78, 1.26) | 0.96 (0.82, 1.19) | 0.92 (0.78, 1.09) | 0.95 (0.81, 1.11) | 0.98 (0.82, 1.17) |
|  | City education*Less than primary | 0.98 (0.73, 1.33) | 0.98 (0.79, 1.21) | 0.98 (0.79, 1.20) | 1.01 (0.82, 1.24) | 0.89 (0.70, 1.13) |
|  | City variance (Std. error) | 0.065 (0.034) | 0.011 (0.013) | 0.029 (0.014) | 0.015 (0.013) | 0.136 (0.042) |
|  | Neighborhood variance (Std. error) | 0.295 (0.110) | 0.159 (0.053) | 0.161 (0.047) | 0.062 (0.039) | 0.315 (0.058) |
| Women |  | *(n = 11,991)* | *(n = 11,991)* | *(n = 12,053)* | *(n = 12,053)* | *(n = 11,913)* |
|  | Individual education |  |  |  |  |  |
|  | University | 1.00 | 1.00 | 1.00 | 1.00 | 1.00 |
|  | Secondary | 1.06 (0.85, 1.33) | 1.15 (1.02, 1.31) | 1.18 (1.03, 1.36) | 1.16 (1.02, 1.32) | 1.09 (0.93, 1.280) |
|  | Primary | 1.31 (1.05, 1.64) | 1.63 (1.43, 1.85) | 1.71 (1.48, 1.97) | 1.49 (1.30, 1.71) | 1.19 (1.00, 1.42) |
|  | Less than primary | 1.57 (1.20, 2.06) | 1.82 (1.53, 2.17) | 2.47 (2.05, 2.98) | 1.10 (0.90, 1.36) | 0.93 (0.69, 1.27) |
|  | Neighborhood education, z-score | 0.87 (0.80, 0.94) | 0.86 (0.81, 0.90) | 0.73 (0.69, 0.77) | 0.99 (0.94, 1.04) | 1.26 (1.18, 1.35) |
|  | City education, z-score | 1.32 (1.09, 1.60) | 1.03 (0.91, 1.16) | 1.15 (0.99, 1.33) | 1.04 (0.91, 1.19) | 1.05 (0.89, 1.24) |
|  | City education * Individual education |  |  |  |  |  |
|  | City education*University | 1.00 | 1.00 | 1.00 | 1.00 | 1.00 |
|  | City education*Secondary complete | 0.81 (0.65, 1.01) | 0.89 (0.79, 1.01) | 0.91 (0.79, 1.05) | 1.06 (0.93, 1.20) | 1.09 (0.93, 1.27) |
|  | City education*Primary complete | 0.87 (0.71, 1.07) | 0.96 (0.85, 1.08) | 0.94 (0.82, 1.08) | 1.04 (0.91, 1.18) | 1.00 (0.84, 1.18) |
|  | City education*Less than primary | 0.73 (0.57, 0.92) | 0.93 (0.79, 1.08) | 1.01 (0.85, 1.19) | 1.06 (0.88, 1.27) | 1.05 (0.80, 1.39) |
|  | City variance (Std. error) | 0.023 (0.018) | 0.040 (0.016) | 0.074 (0.025) | 0.065 (0.022) | 0.093 (0.033) |
|  | Neighborhood variance (Std. error) | 0.150 (0.088) | 0.111 (0.036) | 0.145 (0.039) | 0.039 (0.037) | 0.230 (0.065) |

Interaction model a: include interaction term individual education*city education. Multilevel structure: individuals nested within neighborhoods, nested within cities. Analyses adjusted by age, and total city population. Neighborhood education: proportion of the population aged 25 or older who completed secondary education or above. City education: proportion of the population aged 25 or older who completed secondary education or above. Global p for interactions were not statistically significant. Std. error corresponds to the standard error of the random component of cities and neighborhoods.

Table S2. Odds ratios of non-communicable disease risk factors by individual and neighborhood education and city education. Argentina, National Survey of Risk Factors 2013 (n= 21,451); interaction model b.  

|  |  | **Diabetes** | **Hypertension** | **Obesity** | **Smoking** | **Binge drinking** |
| --- | --- | --- | --- | --- | --- | --- |
|  |  | **OR (95% CI)** |  |  |  |  |
| Men |  | *(n = 9,346)* | *(n = 9,295)* | *(n = 9,398)* | *(n = 9,398)* | *(n = 9,351)* |
|  | Individual education |  |  |  |  |  |
|  | University | 1.00 | 1.00 | 1.00 | 1.00 | 1.00 |
|  | Secondary complete | 1.19 (0.93, 1.54) | 1.26 (1.07, 1.49) | 1.35 (1.14, 1.59) | 1.33 (1.14, 1.56) | 1.12 (0.95, 1.33) |
|  | Primary complete | 1.06 (0.82, 1.38) | 1.42 (1.20, 1.69) | 1.55 (1.30, 1.84) | 1.99 (1.69, 2.34) | 1.73 (1.45, 2.07) |
|  | Less than primary | 1.07 (0.77, 1.49) | 1.18 (0.94, 1.48) | 1.62 (1.29, 2.02) | 1.87 (1.51, 2.33) | 1.59 (1.24, 2.03) |
|  | Neighborhood education, z-score | 0.93 (0.85, 1.02) | 0.98 (0.92, 1.04) | 0.87 (0.82, 0.93) | 0.97 (0.92, 1.02) | 0.97 (0.91, 1.04) |
|  | City education, z-score | 1.06 (0.94, 1.20) | 1.03(0.96, 1.11) | 1.01 (0.93, 1.09) | 1.05 (1.00, 1.10) | 1.09 (0.95, 1.25) |
|  | City education*Neighborhood education | 0.98 (0.90, 1.06) | 1.03 (0.98, 1.09) | 0.95 (0.91, 1.00) | 0.99 (0.95, 1.04) | 1.00 (0.95, 1.06) |
|  | City variance (Std. error) | 0.064 (0.034) | 0.020 (0.012) | 0.030 (0.014) | 0.004 (0.007) | 0.136 (0.042) |
|  | Neighborhood variance (Std. error) | 0.296 (0.110) | 0.156 (0.053) | 0.159 (0.047) | 0.059 (0.039) | 0.313 (0.058) |
| Women |  | *(n = 11,991)* | *(n = 11,991)* | *(n = 12,053)* | *(n = 12,053)* | *(n = 11,913)* |
|  | Individual education |  |  |  |  |  |
|  | University | 1.00 | 1.00 | 1.00 | 1.00 | 1.00 |
|  | Secondary | 1.03 (0.823, 1.28) | 1.15 (1.01, 1.30) | 1.18 (1.02, 1.35) | 1.16 (1.02, 1.32) | 1.11 (0.95, 1.30) |
|  | Primary | 1.27 (1.02, 1.58) | 1.62 (1.42, 1.84) | 1.70 (1.47, 1.96) | 1.50 (1.30, 1.72) | 1.20 (1.01, 1.44) |
|  | Less than primary | 1.54 (1.17, 2.01) | 1.82 (1.52, 2.17) | 2.44 (2.02, 2.93) | 1.11 (0.90, 1.36) | 0.94 (0.70, 1.28) |
|  | Neighborhood education, z-score | 0.87 (0.81, 0.95) | 0.85 (0.81, 0.90) | 0.73 (0.69, 0.77) | 0.99 (0.94, 1.04) | 1.26 (1.18, 1.35) |
|  | City education, z-score | 1.12 (1.02, 1.23) | 0.97 (0.90, 1.06) | 1.08 (0.98, 1.20) | 1.08 (0.98, 1.19) | 1.09(0.96, 1.23) |
|  | City education*Neighborhood education | 0.98 (0.91, 1.05) | 1.01 (0.97, 1.06) | 0.93 (0.89, 0.98) | 0.95 (0.91, 0.99) | 1.00 (0.95, 1.06) |
|  | City variance (Std. error) | 0.022 (0.018) | 0.039 (0.015) | 0.075 (0.025) | 0.063 (0.021) | 0.093 (0.033) |
|  | Neighborhood variance (Std. error) | 0.153 (0.088) | 0.111 (0.036) | 0.141 (0.039) | 0.036 (0.036) | 0.227 (0.065) |

Interaction model b: include interaction term neighborhood education*city education. Multilevel structure: individuals nested within neighborhoods, nested within cities. Analyses adjusted by age, and total city population. Neighborhood education: proportion of the population aged 25 or older who completed secondary education or above. City education: proportion of the population aged 25 or older who completed secondary education or above. Statistically significant interactions were found for obesity (p < 0.01) and smoking (p < 0.05) in women. Std. error corresponds to the standard error of the random component of cities and neighborhoods.
